# Socioeconomic Context and the Food Landscape in Texas: Results from Hotspot Analysis and Border/Non-Border Comparison of Unhealthy Food Environments

**DOI:** 10.3390/ijerph110605640

**Published:** 2014-05-26

**Authors:** Jennifer J. Salinas, Bassent Abdelbary, Kelly Klaas, Beatriz Tapia, Ken Sexton

**Affiliations:** 1 University of Texas School of Public Health, Epidemiology, Human Genetics and Environmental Sciences (EHGES), Brownsville Regional Campus, UTB Campus- RAHC Building, 80 Fort Brown, Brownsville, TX 78520, USA; E-Mails: bassent.e.abdelbary@uth.tmc.edu (B.A.); ken.sexton@uth.tmc.edu (K.S.); 2 Michael and Susan Dell Center for Healthy Living, University of Texas School of Public Health, Austin Regional Campus, 3445 Executive Center Drive Suite 150, Austin, TX 78731, USA; E-Mail: Kelly.Klaas@uth.tmc.edu; 3 School of Medicine, Family and Community Health, University of Texas Health Science Center San Antonio, Regional Academic Health Center, 2102 Treasure Hills Blvd., Harlingen, TX 78550, USA; E-Mail: tapiab@uthscsa.edu

**Keywords:** food environment, Texas, border, socioeconomics, ethnic concentration

## Abstract

*Purpose*: The purpose of this paper is to describe the food landscape of Texas using the CDC’s Modified Retail Food Environment (mRFEI) and to make comparisons by border/non-border. *Methods*: The Modified Retail Food Environment index (mRFEI (2008)) is an index developed by the CDC that measures what percent of the total food vendors in a census track sell healthy food. The range of values is 0 (unhealthy areas with limited access to fruits and vegetables) to (100—Healthy). These data were linked to 2010 US Census socioeconomic and ethnic concentration data. Spatial analysis and GIS techniques were applied to assess the differences between border and non-border regions. Variables of interest were mRFEI score, median income, total population, percent total population less than five years, median age, % receiving food stamps, % Hispanic, and % with a bachelor degree. *Results*: Findings from this study reveal that food environment in Texas tends to be characteristic of a “food desert”. Analysis also demonstrates differences by border/non-border location and percent of the population that is foreign born and by percent of families who receive food stamps. *Conclusions*: Identifying the relationship between socioeconomic disparity, ethnic concentration and mRFEI score could be a fundamental step in improving health in disadvantage communities, particularly those on the Texas-Mexico border.

## 1. Introduction

Obesity rates in the United States are rising, due largely to an increase in sedentary behaviors combined with an increase in dietary intake of fats, carbohydrates and sugars [1]. Texas is among the states with the highest obesity prevalence in the United States, with 29.2% of adults having a Body Mass Index (BMI) of 30 or above [2]. Less than half of all Texans, adults and children, consume the daily amount of fruits and vegetables recommended by the U.S. Department of Health and Human Services [3]. The rates of obesity in Texas are projected to increase and yet few state policies to improve nutrition to curb these trends have been adopted [2]. 

There is increasing evidence to suggest that the distance that an individual has to travel to a grocery store is linked with his/her intake of healthier foods, such as fresh fruits and vegetables, and that proximity to fast-food outlets is linked with an intake of unhealthy foods [1,4,5]. In the United States, low-income and racial/ethnic minority communities often have more restricted access to grocery stores and a higher concentration of fast food and convenience store outlets [6,7,8]. Moreover, the availability of affordable, quality healthy foods in these “at-risk” neighborhoods tends to be restricted to outlets selling a limited selection of foods that are higher priced and high in fat and calories [4,9]. This is particularly the case on the Texas-Mexico border where obesity is juxtaposed with food insecurity [10]. Moreover, it is the availability of healthy food at grocery stores and convenience stores that is associated with obesity and may explain disparities along the Texas-Mexico border region [1,4,5,10,11,12,13]. This study examines the food environment in Texas making use of the Modified Retail Food Environment Index (mRFEI) [2], an index created by the Centers for Disease Control that scores census tracts by the presence/absence of grocery stores, convenience stores, fast food restaurants and other restaurants, linked to 2010 census tract-level population-level risk factors. The purpose of this paper is three-fold: (1) to describe the food landscape of Texas, including a “hot spot” analysis; (2) to compare border and non-border food environments within the State; and (3) to identify population-level risk factors associated with less healthful food environments, contrasting border with non-border communities. 

## 2. Experimental Section

### 2.1. Data

#### 2.1.1. The Modified Retail Food Environment (mRFEI) 2008

The mRFEI is a measure of the percent of total number of food vendors (healthy and less healthy) that are healthy in a census tract. Definitions were based on North American Industry Classification Codes (NAICS) in 2008. The definition of healthy *vs.* unhealthy is defined by typical food offerings such as a grocery store, convenience store and a fast food restaurant. Healthy food vendors were supermarkets, larger grocery stores, supercenters, and fruit and vegetable stands in census tracts or within a 1/2 mile. Supermarkets were defined as having 50 or more employees and large grocery stores were defined as having 10–49 employees. Fruit and vegetables stands were considered vendors that sold fresh produce in a market or a permanent stand. Less healthy food vendors included convenience stores, fast food restaurants, and small grocery stores (three employees or less) in the census tract or within 1/2 mile. The measure does not include dollar stores or similar type retail chains. The mRFEI represents the percent of all the food retailers in a given census tract that are healthy. The potential score for the mRFEI ranges from 0 or “food desert” (e.g., no healthy food vendors) to 100 “healthy” (only healthy food vendors). 

#### 2.1.2. U.S. Census 2010

The census tract data were obtained through the United States Census Bureau, the American fact finder website [14]. Demographic and social characteristics tables were selected and downloaded in a delimited format together with the annotation file. The tables were screened and variables of interest were selected and merged into one file in excel format. A database was then created with the census tract Federal Information Processing Standards (FIPS) codes as our ID indicator. Variables used for this analysis were total population, median age, percent below the poverty line, percent adults 25+ with a high school diploma, percent foreign born, percent Hispanic, and percent of families on food stamps.

#### 2.1.3. Border/Non-Border

In 1983 the Environmental Protection Agency (EPA) signed an agreement with Mexico's Secretariat of Environment and Natural Resources (SEMARNAT) in an attempt to address binational environmental issues that affected both the US and Mexico sides of the border. They defined the border as being the approximately 2000 miles that stretches from the Gulf of Mexico in Texas to the Pacific Ocean in California [15], and 62.5 miles (approx. 100 km) into either country. According to the Article 4 of the La Paz Agreement, a county is considered a border county if any portion is within the 62.5 miles from the Mexico border [16]. Counties were coded as “1” if they were within the 62.5 miles limit and “0” if they were outside. 

#### 2.1.4. Procedure

**Figure 1:**
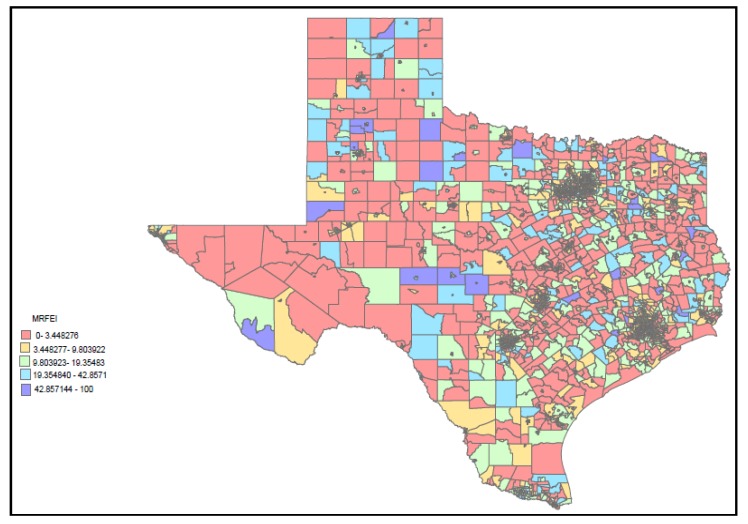
mRFEI scores by census tract for the State of Texas.

Data from the mRFEI were merged with the U.S. Census data matching on the FIPS number, giving a total of 2,512 matched census tracts in Texas that were used in our analysis. Figure 1 provides a summary of average mRFEI scores across the State of Texas. What is most notable about this map is the large number of census tracts with low scores or zeros (e.g., food deserts). These census tracts are likely to be highly rural areas where food options that are healthy or unhealthy are limited or non-existent. It is for this reason that we conducted regression analyses with and without census tracts with mRFEI scores of zero. In addition, there were eight census tracts that were outliers with scores of 75 or above. This is likely due to the fact that there were very few food outlets in these tracts and mostly likely to be grocery stores. These census tracts were also excluded from the analysis to prevent any bias or skewing that may occur as a result of these few census tracts. Skewness analysis was conducted before and after the removal of these census tracts and by taking these tracts out we were able to ensure a normal distribution and thereby comply with assumptions made when using linear regression. 

Descriptive statistics were generated for all census tracts and by border/non-border categories for the average mRFEI Score, total population, median age, percent below the poverty line, percent adults 25+ with a high school diploma, percent foreign born, percent Hispanic, and percent of families on food stamps. We performed linear regression using the jackknife variance estimate to account for clustering of adjacent census tracts and to adjust for the spatial autocorrelation. Interaction models were generated between border/non-border and census tract socioeconomic variables. Finally, data were imported into ARCGIS, where hot spot analysis was conducted for significant spatial clusters of high values (hot spots) and low values (cold spots). 

## 3. Results and Discussion

### 3.1. Results

Table 1 presents statistics for census tract socioeconomic variables and average mRFEI scores for the total census tracts and then by border/non-border. The average overall mRFEI score was 11.3, the average census tract size was about 5,000 people and the median age was 34.9 years. Percent of the population living at or below the poverty line was 19.9% and on average 76.8% of census tract adults had at least a high school diploma. Overall, 16.6% of census tract residents were foreign born and 39.4% were Hispanic. Finally, about 13.7% of families across all census tracts in the State were on food stamps. When we broke down these numbers by border/non-border there are statistically significant differences in average mRFEI score (border 12.9 *vs*. non-border 11.1, *p <* 0.001), median age (border 32.9 years, non-border 35.1 years. *p <* 0.001), percent of families living below the poverty line (border 32.0%, non-border 18.6%, *p <* 0.001), percent foreign born (border 25.7%, non-border 15.6%, *p <* 0.001), percent Hispanic (border 85.4%; non-border 34.6%, *p <* 0.001) and percent of families on food stamps (border 27.1%; non-border 12.2%, *p <* 0.001). 

**Table 1:** Variable Means for Texas.

Census Tract Variables	Total	Border	Non-Border
Average mrFEI Score (%) (mean ± S.D.)	11.3 (7.4)	12.9 (7.6)	11.1 (7.4) ***
Total Population (%) (mean ± S.D.)	4,821.3 (2,085.9)	4,847.1 (2,279.8)	4,818.6 (2,064.9)
Median Age (%) (mean ± S.D.)	34.9 (6.5)	32.9 (5.2)	35.1 (6.6) ***
Percent below Poverty (%) (mean ± S.D.)	19.9 (13.2)	32.0 (14.0)	18.6 (12.4) ***
Percent with a High School Diploma (mean ± S.D.)	76.8 (15.8)	63.9 (15.6)	78.1 (15.2) ***
Percent Foreign Born (%) (mean ± S.D.)	16.6 (12.4)	25.7 (10.5)	15.6 (12.2) ***
Percent Hispanic (%) (mean ± S.D.)	39.4 (28.9)	85.4 (15.0)	34.6 (25.6) ***
Percent Families on Food Stamps (%) (mean ± S.D.)	13.7 (10.8)	27.1 (13.3)	12.2 (9.4) ***

*** denotes significant differences between border and non-border at the *p* = 0.001.

Table 2 presents regression analysis using jackknife variance estimate to account for clustering of adjacent census tracts and spatial autocorrelation. Analysis was conducted for all census tracts and those without mRFEI scores of zero. Beginning with the analysis that includes all census tracts, border census tracts have on average higher mRFEI (3.2 (*p =* 0.000). Other socioeconomic conditions associated with an increase in mRFEI include total population (0.00025 (*p =* 0.002) and median age (0.063 (*p =* 0.035)). Socioeconomic conditions associated with decrease in mRFEI score include percent below poverty line (−0.051 (*p =* 0.007) and percent with a high school diploma (−0.088 (*p =* 0.000)). Percent foreign born, percent Hispanic and percent of families on food stamps were not significantly associated with census tract mRFEI scores. When taking out the census tracts with zero-value mRFEI scores the associations change slightly. Census tracts in a border county on average have mRFEI scores of 4.97 (*p =* 0.000) more than those in non-border counties. For every one year increase in residents’ median age in a census tract, the mRFEI score increases by 0.078 (*p =* 0.011), however the other socioeconomic variables are negatively associated with mRFEI. For example an increase in percent of families who live below the poverty line is associated with 0.076 (*p =* 0.000) decline in mRFEI score. Similarly percent with a high school diploma (β = −0.210, *p =* 0.000), percent foreign born (β = −12.9, *p =* 0.000), percent Hispanic (β = −5.03, *p =* 0.000) and percent families on food stamps (β = −7.99, *p =* 0.00) are all associated with lower mRFEI average scores at a census tract level.

**Table 2:** Regression analysis for mRFEI by border status and socioeconomic conditions using jackknife variance estimate.

Heading	With zero values		Without zero values	
	Coefficient [95% C.I.]	*p*-value	Coefficient [95% C.I.]	*p*-value
Border County (yes = 1)	3.2 [1.8, 4.6]	0.000	4.97 [3.7, 6.2]	0.000
Total Population	0.00025 [0.00009, 0.0004]	0.002	0.000015 [−0.0002, 0.0002]	0.868
Median Age	0.063 [0.004, 0.12]	0.035	0.078 [0.02, 0.13]	0.011
Percent below Poverty	−0.051 [−0.09, −0.01]	0.007	−0.076 [−0.11, −0.04]	0.000
Percent with a High School Diploma	−0.088 [−0.13, −0.05]	0.000	−0.210 [−0.25, −0.17]	0.000
Percent Foreign Born	−2.23 [−5.2, 0.78]	0.146	−12.9 [−15.8, −9.9]	0.000
Percent Hispanic	−1.50 [−3.4, 0.41]	0.125	−5.03 [−6.8, −3.2]	0.000
Percent Families on Food Stamps	−1.37 [−5.9, 3.2]	0.555	−7.99 [−12.2, −3.8]	0.000

Separate interaction models (results not shown) were conducted between border/non-border and percent of families living at or below the poverty line; percent of adults 25+ with a high school diploma; percent Hispanic; percent of families on food stamps and percent foreign born. There were significant interaction effects only for percent of living below the poverty line; percent of families on food stamps; and percent foreign born. Beginning with percent living below the poverty line, the coefficient for the interaction term was 0.095 (*p =* 0.008). The interaction coefficient for families on food stamps was 8.88 (*p =* 0.041) and percent foreign born was 15.9 (*p =* 0.001).

**Figure 2:**
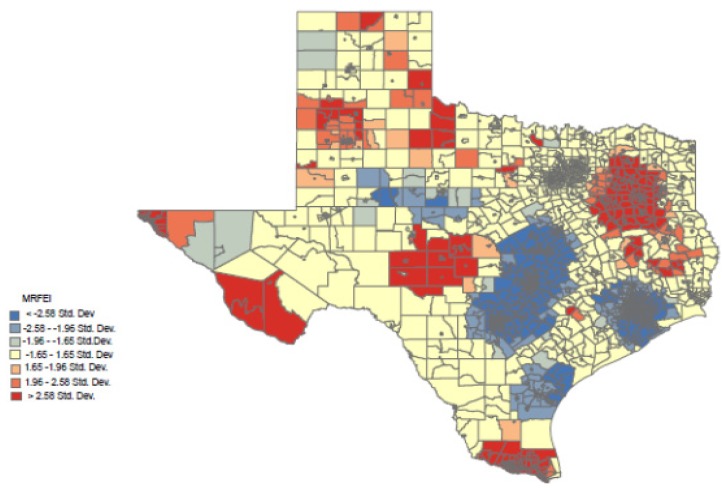
The Hot Spot Analysis (Getis-Ord Gi*).

To further illustrate the relationship between border/non-border status and mRFEI, Figure 2 presents a hot-spot map for the State of Texas. Red is indicative of a higher cluster of higher mRFEI scores, whereas blue represents higher clusters of lower mRFEI scores. Red clusters can be seen along the US-Mexico border region metropolitan areas of Brownsville/McAllen, Laredo and El Paso. A red cluster can also be observed in the Tyler metropolitan area. Blue clustering can be observed in the San Antonio and Austin metropolitan areas of Central Texas, Corpus Christi and the Houston metropolitan area. While overall mRFEI scores are low, the census tracts with the lowest scores appear to be clustered in three major metropolitan areas of Texas and not on the border. 

### 3.2. Discussion

The purpose of this study was to characterize the food environment in the State of Texas and to determine differences by border/non-border status in food environment and the relationship between socioeconomic and food environments. Findings from this study reveal that overall the food environment in Texas tends to be characteristic of a “food desert” according to CDC definition [2]. Even in larger metropolitan areas like Austin or Houston the food environment is not conducive to nutritional behavioral change to reduce obesity. Additionally, while border region is often characterized as food insecure [9], analysis for border/non-border status reveals that census tracts in the U.S.-Mexico border region on average have better food environments than non-border. The interaction models by border status suggest that socioeconomic conditions such as percent of the population that is foreign born or percent of families who receive food stamps may not translate into poorer food environments necessarily, but depend also on where the census tract is geographically located. 

Smaller-scaled studies that have looked at food environment have observed differences by race/ethnic concentration and socioeconomics [17,18,19,20]. Poorer and ethnic minority communities in metropolitan areas tend to have worse food environments than more affluent and non-Hispanic white communities [21,22,23]. However, in a large geographic area, like Texas, the differences are more complex. The overall food environment in Texas does not appear to be conducive to good nutritional behaviors recommended by the U.S. Department of Health & Human Services and the federal government [3]. Many census tracts had mRFEI scores that were zero. These census tracts are most likely to be rural areas that do not have ready access to any health promoting resource in general or lack of transportation options play an essential role in rural communities limiting access to healthy food and health services. 

#### 3.2.1. Food Access an Issue on the US-Mexico Border

Food access on the US-Mexico border has been documented as challenging for its residents; as fresh fruit and vegetables are not readily available forcing residents travel further to obtain healthy food [6]. The US-Mexico border regions, populated primarily by persons of Mexican origin, are known to have increasing population-level obesity rates that are much higher than the US overall [9]. Research on the border environment suggests that food insecurity is closely linked to food access [20]. Socioeconomic status is strongly and inversely related to grocery store proximity and availability, and residents who live in lower-income and racial/ethnic minority border towns have to travel a significantly greater distance to the closest grocery store [8,9,10,23]. Many residents in poorer areas of the border region are relying on dollar stores and convenience store as food providers [23]. Moreover, dollar stores are often utilized as the main resource for fruits and vegetables [24]. Nevertheless, many border towns lack adequate roads, having limited or no public transportation and experience poor access to community resources overall, thereby illustrating the complexity of food access in the US-Mexico border region [4]. The findings from this study provide initial evidence that the border food environment may be differ in substantive ways from other areas of Texas. It is unclear if one or two percentage points may be significant enough to conclude any causal relationship between food environment and obesity prevalence. There have been few studies that have evaluated the clinic significance of such small differences in large-scale instruments such as the mRFEI, and therefore a direction for future research. Additionally, further investigation is warranted to determine to what extent the socioeconomic conditions vary by border/non-border status and this might influence the types of food vendors that sell healthier foods. 

#### 3.2.2. Quality not Quantity

Findings from this study suggest that the food environment on the U.S.-Mexico border may be slightly better than that found in metropolitan areas of Texas, like Austin, San Antonio and Houston. Even taking into consideration food stamps, poverty and foreign born, in similar census tracts, the food environment on the US-Mexico border, on average, tends to have a higher percentage of food vendors that offer healthier foods than in non-border areas of Texas. The mRFEI is based on the percentage of food outlets in a census tract that are healthy, therefore it may be that border census tracts may have a greater percentage of more healthy food outlets, but a lower overall number of food sources. Therefore solutions to food environment issues in the border region may be more centered on transportation, while solutions in non-border areas may focus on increasing the percentage of healthy food outlets relative to unhealthy food outlets. 

#### 3.2.3. The Socioeconomics of Food Access

Socioeconomic factors that were evaluated in this study included percent of families living at or below the poverty line, adults 25+ years with at least a high school diploma, percent families receiving food stamps, percent Hispanic and percent foreign born. In all cases, as the percentage increased, the mRFEI score decreased. This is consistent with findings from other studies that have established a negative relationship between socioeconomic conditions of a community and the local food environment. Dubowitz *et al*. [6] used NHANES data to show that in neighborhoods -measured as census tracts- the availability of fruits and vegetables was greater in affluent than in less affluent settings. In addition, findings from a New Zealand study suggest that residents of low income neighborhoods have to travel further for healthier food, but less time for fast food that those in higher income neighborhoods [25]. The current study provides added support for the association of socioeconomics and food environment in Texas, and suggests a possible opportunity to improve health through policies directed at making healthy food more accessible in low-income neighborhoods and/or providing better transportation systems that facilitate easier access to healthier food outside of neighborhoods. 

#### 3.2.4. Study Limitations

This study provides information on the overall food environment of the state of Texas, including a hot spot analysis and a comparison between border and non-border areas. The findings from this study are novel, yet limitations must be taken into account when interpreting our results. First, this study is cross sectional and therefore we are only about to infer associations and not causation. Second, since these data are aggregated to census tract level, findings are not necessarily applicable to individual behaviors and, therefore, should be considered valid only at a census tract level. Third, the mRFEI instrument is a percentage of overall food outlets that are healthy as defined by the CDC definition, which does not take into consideration the density of food outlets in general, therefore making it difficult to glean the impact of urban *vs*. rural issues of food environment as compared to food access overall. Moreover, there are many other sources of food outlets, as mention earlier, such as dollar stores that are increasingly becoming destinations for both healthy and unhealthy foods. Finally, we conduct our analysis using jackknife variance estimate and hot spot analyses, which are just two techniques to evaluate data like these. Alternative approaches may yield differential findings than what we have observed in this study. 

## 4. Conclusions

This study provides needed information on geographic and socioeconomic patterns in food environment that can help direct efforts to improve food environments across Texas and the United States and contribute to an overall decline in obesity. Texas is among the states with the highest prevalence of obesity in the country. Understanding socioeconomic contextual risk factors for obesity such as food environment are important directions to take in order to create policy and targeted efforts to reduce the prevalence of obesity such as modifying food environments that may put populations at risk for obesity. The obesity epidemic has reached global proportions with one in three adults now being obese [1]. Resolving this crisis may require focusing on both individual behavior and food environments that serve as barriers to nutritional behavior change.
